# Supplementary material for: Hepatitis C Virus Epidemiology in Lithuania: Situation before Introduction of the National Screening Programme
Source: Viruses. 2022 May 30;14(6):1192. doi: 10.3390/v14061192 (PMC9230764; doi:10.3390/v14061192)
Supplement: Supplementary file 1 [file viruses-14-01192-s001.zip › viruses-1685482-supplementary.pdf]

Table S1. Sex-stratified and total annual age-standardized mortality rates per 100 000 people for chronic viral hepatitis C (CHC) as underlying cause, unspecified liver cirrhosis and hepatocellular carcinoma with CHC as a contributory cause.

Year	Causes of death (ICD-10 codes)														
	CHC (B18.2) (underlying cause)					Unspecified liver cirrhosis with CHC as a contributory cause (K74.6 + B18.2)					Hepatocellular carcinoma with CHC as a contributory cause (C22.0+B18.2)				
	Men		Women		Total	Men		Women		Total	Men		Women		Total
	n	Rate	n	Rate	Rate	n	Rate	n	Rate	Rate	n	Rate	n	Rate	Rate
2010	13	1.07	5	0.28	0.59	5	0.38	3	0.16	0.27	0	0	2	0	0
2011	14	1.02	9	0.47	0.75	5	0.39	1	0.05	0.20	3	0.28	3	0.12	0.19
2012	6	0.44	8	0.41	0.45	7	0.56	2	0.12	0.30	4	0.28	1	0.18	0.23
2013	19	1.46	8	0.45	0.90	5	0.34	2	0.12	0.22	3	0.24	3	0.04	0.13
2014	16	1.16	10	0.56	0.86	5	0.34	2	0.12	0.23	3	0.21	3	0.17	0.20
2015	17	1.25	10	0.60	0.92	3	0.25	3	0.18	0.20	7	0.60	3	0.15	0.34
2016	15	1.20	10	0.55	0.84	10	0.76	3	0.19	0.47	10	0.74	3	0.18	0.43
2017	13	0.94	8	0.49	0.75	7	0.60	4	0.25	0.38	2	0.18	3	0.17	0.17
2018	14	1.13	9	0.52	0.83	8	0.59	2	0.11	0.33	10	0.78	3	0.18	0.43
2019	17	1.36	5	0.36	0.77	8	0.62	6	0.34	0.49	6	0.54	3	0.16	0.30
2020	11	0.81	5	0.26	0.54	7	0.52	1	0.07	0.29	14	1.03	2	0.18	0.56
